# Synthesis and Performance Evaluation of a High-Temperature-Resistant Plugging and Inhibition Agent

**DOI:** 10.3390/molecules31132288

**Published:** 2026-07-01

**Authors:** Yue Gao, Cheng Ma, Xuan Qi, Hao Yan, Nadiremu Kamaliding, Junfeng Zhang

**Affiliations:** 1 College of Chemical and Materials Engineering, Hainan Vocational University of Science and Technology, Haikou 571126, China; 2 School of Petrchemical Engineering, Liaoning Petrochemical University, Fushun 113001, China; 3 School of Chemistry and Chemical Engineering, Hainan University, Haikou 570228, China

**Keywords:** emulsion polymerization, nanoparticles, plugging–inhibitor, high-temperature stability, filtration loss control

## Abstract

A high-temperature-resistant plugging–inhibitor (DS) was synthesized via emulsion polymerization using styrene (St), butyl acrylate (BA), 2-acrylamide-2-methylpropanesulfonic acid (AMPS), and N-vinylformamide (NVF), with divinylbenzene (DVB) as crosslinker and ammonium persulfate (APS) as the initiator. The structure and properties were characterized by FTIR, TG, SEM, and contact angle analysis. Plugging and inhibition performances were evaluated through filtration, high-temperature high-pressure (HTHP) plugging, linear swelling, and shale recovery tests. DS exhibits spherical morphology with particle sizes of 50–100 nm and a decomposition temperature of ~297 °C, indicating good thermal stability. The addition of DS significantly reduces filtration loss; at 1.5 wt%, the API filtration loss decreases to 6 mL. Under 180 °C conditions, 1 wt% DS reduces HTHP filtration loss from 12 to 6 mL, demonstrating excellent high-temperature plugging performance. DS also effectively suppresses shale hydration, with linear swelling reduced to 1.48 mm and shale recovery increased to 64.68% at 3 wt%. These results indicate that DS possesses excellent thermal stability, plugging efficiency, and inhibition performance, offering a promising solution for wellbore stability in shale gas drilling.

## 1. Introduction

With the rapid development of nanotechnology, nanoscale plugging–inhibitors have emerged as highly effective additives for stabilizing shale wellbores in drilling operations [1,2,3,4]. Conventional drilling fluid additives often fail under high-temperature and high-pressure conditions encountered in deep wells and complex formations. Many nanoparticles have been developed to improve the filtration and rheological properties of water-based drilling fluids by tailoring their type, surface properties, and particle size. These nanoparticles can form a thin and dense filter cake and significantly retard pore pressure transmission, enhancing wellbore stability.

**Inorganic Nano-Protective Materials**

Inorganic nanoparticles such as silica, calcium carbonate, and metal oxides are widely used due to their high stability and adsorption properties. Nano-silica possesses abundant surface hydroxyl groups, which improve suspension stability and rheology, while its spherical microstructure is environmentally friendly [5,6]. For example, Taraghikhah et al. [5] reported that the addition of 1% nano-silica significantly improved plugging efficiency, rheology, lubrication, and shale recovery compared to conventional drilling fluids. Aramendiz et al. [6] showed that co-application of nano-silica and graphene sheets in water-based drilling fluids could disperse within the filter cake’s nanopores, forming a denser and smoother cake, reducing HTHP filtration loss by 27.21%.

Nano-calcium carbonate is another widely used material due to its small particle size, abundance, low cost, and acid solubility [7,8]. Zhang et al. [7] developed nano-CaCO_3_ to enhance drilling fluid bridging and plugging, achieving up to 95.5% reduction in core permeability at 1% addition. Zhang [8] solved dispersion challenges at higher concentrations by using dispersants, resulting in a shale water-swelling rate of only 15% and rolling recovery of 88.6%.

Metal oxide nanoparticles, such as TiO_2_, Fe_2_O_3_, BiFeO_3_, Fe_3_O_4_, and ZnO, have also been proven as environmentally friendly drilling fluid additives, enhancing thermal stability while reducing filtration loss [9,10,11,12,13]. Parizad et al. [10] reported that TiO_2_ incorporation in water-based drilling fluids reduced HTHP filtrate loss by 25% while maintaining 97.2% shale recovery. Perween et al. [11] showed that BiFeO_3_ nanoparticles fill clay interlayers, reducing filtration loss by nearly 50%. Tian et al. [12] and Mahmoud et al. [13] demonstrated similar improvements using modified Fe_3_O_4_ and Fe_2_O_3_ nanoparticles, forming dense filter cakes and enhancing rheological properties.

**Organic Nano-Protective Materials**

Organic nanoparticles provide plasticity, allowing compression and deformation under high-temperature and high-pressure conditions, effectively plugging micro-scale pores. Common types include polymer microspheres, microemulsions, micellar gels, and nanofibers [14,15,16,17,18,19,20,21,22,23,24,25,26].

Polymer microspheres can bridge and seal shale pores to prevent instability. Wang et al. [15] prepared thermo-sensitive polymer nanospheres (~100 nm) that densely pack in micropores and microfractures, forming a tight layer. Liu et al. [16] found that driving flow rate, particle size, and solution concentration influence plugging efficiency.

Microemulsions with nanoscale droplet sizes can rapidly penetrate micro- and nanopores, reduce interfacial tension, and enhance lubrication [17,18,19]. Micellar gels form a 3D polymer network that adsorbs on fracture surfaces and fills pores, improving flow resistance [20,21,22,23]. Nanofibers, such as cellulose nanofibers, offer renewable, biodegradable, and high-strength properties, forming extensive hydrogen-bond networks in drilling fluids to enhance plugging and reduce filtration loss [24,25,26].

**Inorganic–Organic Composite Materials**

Composite nanoparticles combine rigid inorganic cores with flexible organic polymers to achieve high-temperature stability and pore bridging simultaneously. They form dense semi-permeable membranes in shale pores, reducing filtrate invasion and protecting wellbore integrity [27,28]. For example, surface-grafted nano-CaCO_3_ and thermosensitive polymer/nano-SiO_2_ composites have been shown to enhance shear-thinning behavior, reduce filtrate loss, and improve shale stability in high-temperature water-based drilling fluids.

These advances highlight the rationale and novelty of the current work: the DS plugging–inhibitor combines amphoteric charge functionality (NVF-derived groups and anionic AMPS/APS groups) and nanoscale morphology, enabling both nanopore/microfracture plugging and shale hydration inhibition under high-temperature conditions, which distinguishes it from conventional inorganic or organic nanoparticles.

In this study, the DS plugging–inhibitor was synthesized via emulsion polymerization using styrene (St) and butyl acrylate (BA) as the main monomers, with divinylbenzene (DVB) as a crosslinker, ammonium persulfate (APS) as an initiator, and deionized water as the dispersion medium [29,30,31]. The chemical structure, thermal stability, and micro-morphology of the synthesized DS were characterized using FT-IR, thermogravimetric analysis (TG), scanning electron microscopy (SEM), and contact angle measurements.

The performance evaluation included API filtration tests, high-temperature high-pressure (HTHP) plugging experiments, and shale microstructure analysis before and after treatment [32,33,34]. Furthermore, clay hydration inhibition was assessed through linear swelling tests, mud ball immersion, and rolling recovery experiments to validate the applicability of DS in maintaining wellbore stability during shale gas drilling operations [35,36,37]. This approach provides a systematic framework for evaluating flexible nanoscale plugging–inhibitors under both laboratory and field-relevant conditions [38,39,40,41].

## 2. Experimental

### 2.1. Preparation of DS Plugging–Inhibitor

**Figure 1:**
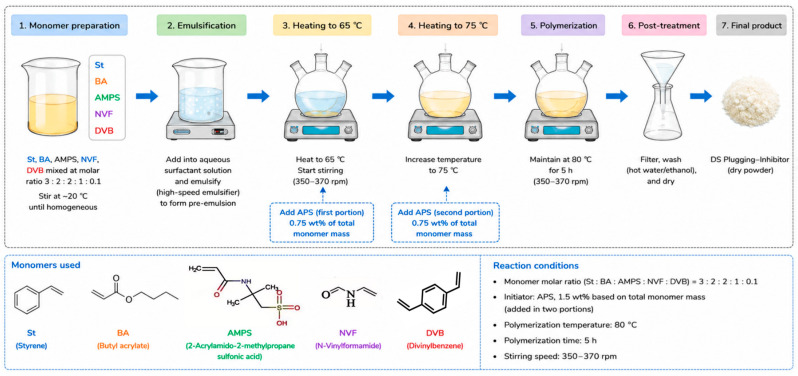
Schematic illustration of the preparation procedure of the DS plugging–inhibitor. The DS plugging–inhibitor was synthesized by emulsion polymerization using St, BA, AMPS, NVF, and DVB (molar ratio = 3:2:2:1:0.1). APS (1.5 wt% of total monomers) was used as the initiator, and polymerization was carried out at 80 °C for 5 h. NVF is included in the reaction to provide neutral amide groups that, together with anionic AMPS/APS units, confer amphoteric charge functionality for interaction with clay.

The DS plugging–inhibitor was synthesized via an emulsion polymerization process (Figure 1). Styrene (St, ≥99%, Aladdin Biochemical Technology Co., Ltd., Shanghai, China), butyl acrylate (BA, ≥99%, Aladdin Biochemical Technology Co., Ltd., Shanghai, China), 2-acrylamido-2-methylpropane sulfonic acid (AMPS, ≥99%, Aladdin Biochemical Technology Co., Ltd., Shanghai, China), N-vinylformamide (NVF, ≥98%, Aladdin Biochemical Technology Co., Ltd., Shanghai, China), and divinylbenzene (DVB, ≥80%, Aladdin Biochemical Technology Co., Ltd., Shanghai, China) were mixed at a molar ratio of 3:2:2:1:0.1. The monomers were stirred at room temperature (~20 °C) until a homogeneous mixture was obtained.

The monomer mixture was slowly added into an aqueous surfactant solution prepared with sodium dodecyl sulfate (SDS, Aladdin Biochemical Technology Co., Ltd., Shanghai, China) and emulsified using a high-speed homogenizer (IKA T25 digital ULTRA-TURRAX, IKA-Werke GmbH & Co. KG, Staufen, Germany) at 10,000–12,000 rpm to form a stable pre-emulsion. Ammonium persulfate (APS, ≥98%, Aladdin Biochemical Technology Co., Ltd., Shanghai, China), used as the initiator at 1.5 wt% of total monomer mass, was added in two stages: the first portion when the system reached 65 °C and the second portion at 75 °C.

Polymerization was conducted in a jacketed glass reactor (500 mL) at 80 °C for 5 h under continuous mechanical stirring (350–370 rpm). Temperature control was maintained using a digital thermostatic water/oil bath (Julabo GmbH, Seelbach, Germany).

After polymerization, the DS plugging–inhibitor latex was obtained, filtered, washed sequentially with hot deionized water and ethanol (analytical grade, Sinopharm Chemical Reagent Co., Ltd., Shanghai, China), and dried under vacuum at 50 °C for 24 h to yield the final product.

### 2.2. API Filtration Test

To investigate the effect of the combined use of the plugging–inhibitor and a conventional fluid loss additive on filtration performance, experiments were conducted using the synthesized plugging–inhibitor in conjunction with bentonite slurry and PAC-LV. A 4 wt% bentonite slurry containing a fluid loss additive was used as the blank control sample.

The plugging–inhibitor was added at concentrations of 1 wt%, 2 wt%, and 3 wt%, respectively. The standard filtration loss was measured over 30 min under medium-pressure conditions for each formulation.

After the filtration tests, the resulting filter cakes were dried and characterized by scanning electron microscopy (SEM). The microstructural morphology of the filter cakes before and after the addition of the nanoparticle plugging agent was analyzed to qualitatively evaluate the plugging performance of the high-temperature-resistant plugging–inhibitor.

Preparation of Freshwater-Based Bentonite Slurry (Base Fluid 2):

Base Fluid 2 was prepared by accurately measuring 1000 mL of deionized water into a clean container. Then, 40 g of bentonite and 2 g of anhydrous sodium carbonate were added. The mixture was stirred at high speed for 20 min. During stirring, the process was paused at least twice to scrape the bentonite adhering to the container walls back into the slurry to ensure complete dispersion. After stirring, the container was sealed and allowed to mature at room temperature for 24 h, resulting in a 2% freshwater-based bentonite slurry. This procedure ensures thorough dispersion of bentonite, providing a uniform base fluid suitable for subsequent addition of the DS plugging–inhibitor.

The rheological properties of the prepared Base Fluid 2 were measured, providing a reference for evaluating the effectiveness of the plugging–inhibitor. The slurry exhibited the following characteristics:

Plastic viscosity (PV): 7.5 mPa·s;

Yield point (YP): 14.3 Pa.

The slurry displayed typical shear-thinning behavior, ensuring good flowability and stability before addition of the inhibitor.

### 2.3. Microstructural Observation of Filter Cake Before and After Plugging

To further verify the plugging performance of the plugging–inhibitor, a 4 wt% bentonite slurry with a fluid loss additive was used as the blank control. API filtration tests were conducted on drilling fluid systems containing 1 wt%, 2 wt%, and 3 wt% plugging–inhibitor, respectively.

After the filtration tests, the filter cakes were collected and their surface morphology and compactness were examined. The plugging performance of the plugging–inhibitor was qualitatively evaluated based on the degree of densification and structural integrity of the filter cake surface before and after the addition of the plugging–inhibitor.

### 2.4. High-Temperature and High-Pressure Sand Bed Plugging Test

The plugging performance of the drilling fluid was evaluated using a high-temperature and high-pressure permeability plugging apparatus (PPA). A circular ceramic sand disk with an average pore size of 3 μm was selected as the filtration medium. The experimental procedure was as follows:

First, the sand disk was immersed in the base fluid for 10 min to achieve full saturation. Subsequently, 350 mL of the test drilling fluid was poured into the cell of the apparatus, and the pretreated sand disk was installed.

The system was then heated to the desired temperature, and a pressure of 3.5 MPa was applied. The filtration volume was recorded over a period of 30 min to evaluate the plugging performance.

For comparison, control experiments were conducted using base fluids without the addition of the plugging–inhibitor under identical conditions.

### 2.5. Linear Swelling Test

The linear swelling behavior was evaluated using bentonite samples dried at 105 ± 3 °C for 4 h and sieved through a 0.15 mm standard sieve. A total of 10.00 g of the treated bentonite was used as the test material. The sample was loaded into the measuring cell of a shale swelling instrument and subjected to a pressure of 4 MPa for 5 min to prepare a standard core sample.

The prepared core, together with the measuring cell, was then installed into the swelling test system. A test solution with a concentration of 3 wt% was introduced, and the swelling variation in the core was continuously recorded over a period of 8 h. For comparison, a blank test using distilled water was conducted under identical conditions.

The inhibition efficiency of clay swelling was calculated using Equation (1):

H = (ΔH_1_ − ΔH)/ΔH_1_ × 100 (1)

where H is the swelling reduction rate (%), ΔH is the swelling height of the core in the 3% test solution (mm), and ΔH_1_ is the swelling height of the core in distilled water (mm). The final results were obtained by averaging two parallel experiments.

It should be noted that deionized water was used to provide a controlled baseline for evaluating clay inhibition. Due to the complexity of drilling fluid systems, this controlled environment allows us to observe the intrinsic inhibition effect of the synthesized DS plugging–inhibitor without interference from other components. The results of this test are further corroborated by the mud ball immersion and shale rolling recovery experiments, which employ realistic drilling fluid matrices.

### 2.6. Mud Ball Immersion Test

(1) Mud Ball Immersion Test at Room Temperature

Bentonite and water were mixed at a mass ratio of 3:1 to prepare uniform mud balls weighing approximately 6 g, with smooth surfaces and no visible cracks. The prepared mud balls were immersed in plugging–inhibitor solutions with different concentrations. The morphology of the mud balls was recorded by photographing them after 24 h of immersion.

(2) Mud Ball Immersion Test under High-Temperature Conditions

Mud balls were prepared following the same procedure described above. The samples were then immersed in plugging–inhibitor solutions with different concentrations and aged at 120 °C for 5 h. After cooling to room temperature, the morphology of the mud balls was recorded by photography.

### 2.7. Shale Rolling Recovery Test

A total of 350 mL of deionized water was added into a high-speed mixing cup, followed by the addition of 10.50 g (accurate to 0.01 g) of bentonite to prepare the base slurry. The mixture was stirred at high speed to ensure complete dispersion. During the process, stirring was stopped at least twice to scrape off any residual material adhering to the cup walls.

Shale samples were dried at 105 ± 3 °C and cooled to room temperature. Subsequently, 50.00 g (accurate to 0.01 g) of shale particles (denoted as M_0_) was added into the prepared slurry. The mixture was then transferred into a rolling aging cell and placed in a roller oven at 150 °C for 16 h.

After rolling, the samples were removed and cooled to room temperature. The shale particles were recovered using 5-mesh and 10-mesh sieves, then dried again at 105 ± 3 °C to constant weight and cooled to room temperature before weighing to obtain the recovered mass (M).

The shale rolling recovery rate (R) was calculated according to Equation (2):

R = M/M_0_ × 100 (2)

where M_0_ is the initial mass of shale (g), M is the recovered mass after rolling (g), and R is the shale recovery rate (%).

## 3. Results and Discussion

### 3.1. Characterization of Plugging–Inhibition Agents

#### 3.1.1. FTIR Analysis

**Figure 2:**
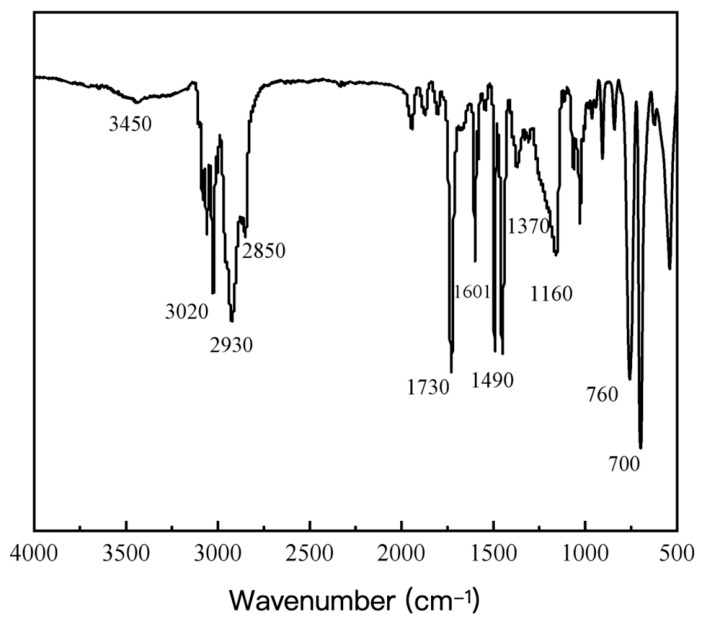
Infrared spectra of occlusion-inhibitor DS.

Figure 2 presents the Fourier transform infrared (FTIR) spectrum of the DS sample. The spectral analysis reveals several characteristic absorption bands corresponding to specific functional groups. A broad peak observed around 3450 cm^−1^ is attributed to the N–H stretching vibration of amine groups. The absorption bands at 3020 cm^−1^ and 2930 cm^−1^ correspond to the stretching vibrations of unsaturated and saturated C–H bonds, respectively.

A distinct peak at 1730 cm^−1^ is assigned to the stretching vibration of the amide carbonyl (C=O) group. The characteristic bands at 1601 cm^−1^ and 1490 cm^−1^ are associated with the skeletal vibrations of the aromatic ring. Additionally, the absorption at 1380 cm^−1^ confirms the presence of the amide C–N bond. The peaks located at 1160 cm^−1^, 760 cm^−1^, and 700 cm^−1^ are attributed to the out-of-plane bending vibrations of aromatic C–H bonds.

The inhibition behavior of the plugging–inhibitor is attributed to the synergistic effects of nanoscale physical plugging and molecular adsorption. The polymer nanoparticles can penetrate into shale nanopores and microfractures, thereby reducing fluid invasion pathways. Meanwhile, the amide groups originating from NVF are capable of forming hydrogen bonds with hydroxyl groups located on clay mineral surfaces, enhancing polymer adsorption. In addition, the styrene and butyl acrylate segments provide hydrophobic characteristics that reduce water uptake by the shale matrix. These combined effects suppress clay hydration and improve wellbore stability.

It should be noted that the NVF units remain in the form of nonionic formamide groups after polymerization. No post-polymerization hydrolysis treatment was performed during synthesis; therefore, vinylamine-type groups are not expected to be present in the polymer structure. Consequently, the interaction between the polymer and clay minerals is primarily attributed to hydrogen bonding and adsorption effects rather than electrostatic attraction arising from protonated amine groups.

#### 3.1.2. TG Analysis

**Figure 3:**
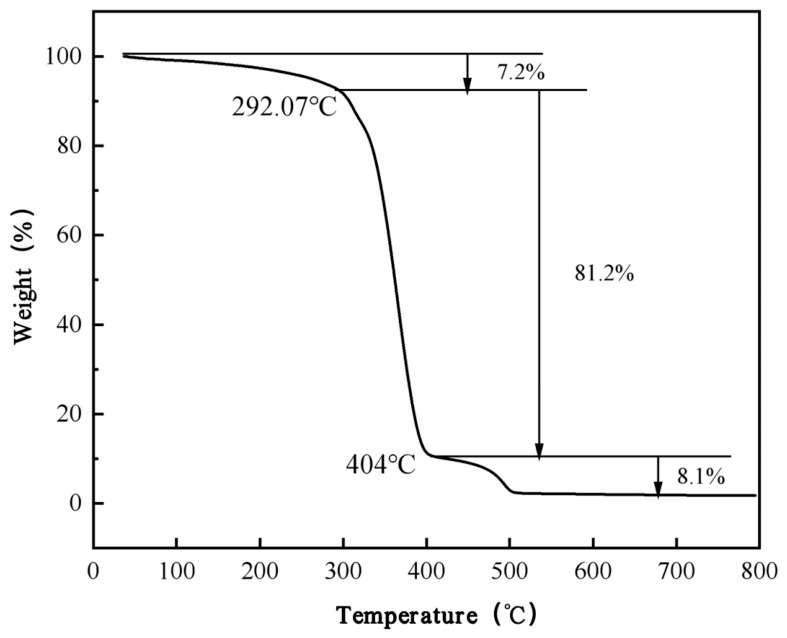
Thermogravimetric curves of occlusion-inhibitor DS.

Figure 3 illustrates the thermogravimetric (TG) curve of the plugging–inhibition agent. The thermal degradation process can be divided into three distinct stages. The first stage occurs from room temperature to 292.07 °C, during which the weight loss is approximately 7.2%. This mass reduction is primarily attributed to the evaporation of free water present in the plugging–inhibition agent powder.

The second stage spans from 292.07 °C to 404 °C and exhibits a significant weight loss of 81.2%, which is mainly due to the decomposition of the polymer backbone. The third stage occurs between 404 °C and 502 °C, with a weight loss of 8.1%, corresponding to the carbonization of the polymer structure.

Beyond 517 °C, the mass of the material remains nearly constant, indicating the completion of thermal decomposition. These results demonstrate that the plugging–inhibition agent undergoes minimal mass loss below 290.07 °C, suggesting that it possesses good thermal stability within this temperature range.

As shown in Table 1, the plugging–inhibitor exhibits excellent thermal stability at elevated temperatures. The temperature corresponding to a 10% mass loss is 307.1 °C, while 30% and 50% mass losses occur at 346.1 °C and 362.1 °C, respectively. These results indicate that the plugging–inhibitor maintains structural integrity over a relatively wide temperature range before undergoing significant thermal degradation. In addition, the residual char yield of the plugging–inhibitor is 1.776%, suggesting limited solid residue formation after thermal decomposition. Overall, the thermal analysis demonstrates that the plugging–inhibitor possesses favorable high-temperature resistance, making it suitable for applications under harsh thermal conditions.

#### 3.1.3. SEM Analysis

**Figure 4:**
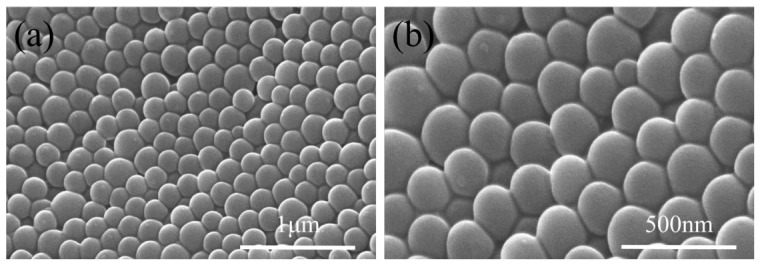
SEM images of the DS plugging-inhibitor at different magnifications. (**a**) Low-magnification image showing the overall spherical morphology and uniform dispersion of the nanoparticles; (**b**) high-magnification image revealing the particle size in the range of 50-100 nm.

The microstructural morphology of the high-temperature-resistant nanopolymer plugging agent was characterized by scanning electron microscopy (SEM). A 1 wt% plugging–inhibitor emulsion was deposited onto conductive adhesive and dried prior to observation using a Regulus-8200 field-emission SEM. The SEM images of the DS plugging–inhibitor at different magnifications are presented in Figure 4.

As shown in Figure 4, the DS plugging–inhibitor particles exhibit a well-defined spherical morphology with a relatively uniform size distribution. The particle size is predominantly in the range of 50–100 nm, confirming the successful preparation of nanoscale materials. Moreover, the particles are evenly dispersed within the system, with no evident aggregation observed.

These results demonstrate that the synthesized DS plugging–inhibitor not only meets the nanoscale size requirements but also possesses excellent dispersion stability. Such structural characteristics are expected to provide a solid foundation for its superior plugging performance, particularly under complex conditions.

#### 3.1.4. Contact Angle Analysis

The wettability of the bentonite pellet surface was quantitatively evaluated using contact angle measurements. In the experiment, bentonite pellets with a thickness of 5 mm were immersed in deionized water and DS plugging–inhibitor solutions of various concentrations for 24 h. After drying, the contact angles were measured using an optical contact angle analyzer. During the measurement, a 5 μL droplet of deionized water was carefully deposited onto the pellet surface using a microsyringe, and the contact angle was recorded after equilibration for 5 min.

The results indicate that the untreated bentonite pellet exhibits strong hydrophilicity, with a contact angle of only 13.0°. This behavior can be attributed to the intrinsic hydrophilic nature of clay minerals in bentonite, where surface hydroxyl groups (–OH) readily form hydrogen bonds with water molecules, resulting in pronounced hydration.

In contrast, after treatment with the DS plugging–inhibitor solution, the surface wettability of the bentonite pellet is significantly altered, as evidenced by the increased contact angle. This change can be explained by the adsorption of inhibitor molecules onto the clay surface via electrostatic interactions. The hydrophilic groups anchor onto the surface, while the lower hydrophilic tails orient outward, forming a lower hydrophilic molecular layer. This layer effectively reduces the direct contact between water molecules and the clay surface.

**Figure 5:**
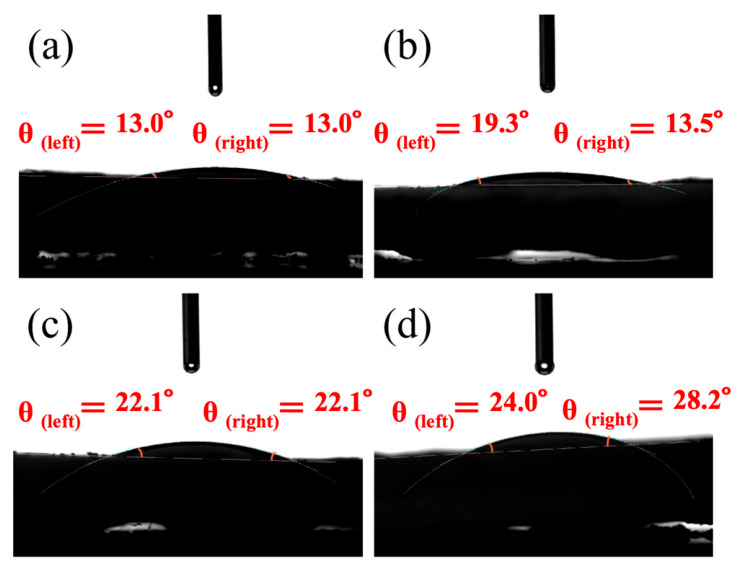
Contact angle of bentonite pellets after immersion in (**a**) deionized water, (**b**) 1 wt% DS plugging–inhibitor solution, (**c**) 2 wt% DS plugging–inhibitor solution, and (**d**) 3 wt% DS plugging–inhibitor solution, showing the reduction in surface hydrophilicity after treatment.

These results demonstrate that the DS plugging–inhibitor reduces the hydrophilicity of the bentonite pellet surface through interfacial molecular modification. Moreover, the results provide direct experimental evidence for the mechanism by which the plugging–inhibitor suppresses the hydration and swelling of bentonite (Figure 5).

### 3.2. Evaluation of Plugging Performance of DS Plugging–Inhibitor

#### 3.2.1. API Filtration Test

To investigate the effect of the combined use of the plugging–inhibitor and fluid loss additive on filtration performance, a series of experiments were conducted using the plugging–inhibitor. A 4 wt% bentonite slurry with a fluid loss additive was selected as the blank control sample. The plugging–inhibitor was added at concentrations of 1 wt%, 2 wt%, and 3 wt% to evaluate its influence on filtration behavior. The standard filtration volume was measured over 30 min for each formulation. In addition, the filter cakes obtained after the filtration tests were dried and subsequently characterized by scanning electron microscopy (SEM). The microstructural morphology of the filter cakes before and after the addition of the nanoparticle plugging agent was analyzed to qualitatively assess the plugging performance of the DS plugging–inhibitor. These results provide insights into the synergistic effects between the plugging–inhibitor and fluid loss additive, as well as the underlying mechanisms governing filtration reduction and pore-sealing behavior.

Notes for Base Fluid 2:

It was prepared by dispersing 40 g bentonite and 2 g anhydrous sodium carbonate in 1000 mL deionized water. The solution was stirred at high speed for 20 min, and paused at least twice to scrape bentonite from container walls. It matured at room temperature for 24 h. The rheological properties are PV = 7.5 mPa·s and YP = 14.3 Pa. It serves as the base for DS plugging–inhibitor evaluation.

As shown in Table 2, the concentration of the DS plugging–inhibitor has a significant effect on the rheological properties of the water-based drilling fluid. Under non-aged conditions, the apparent viscosity (AV), plastic viscosity (PV), and yield point (YP) all exhibit an increasing trend with increasing inhibitor concentration. After aging at 150 °C for 16 h, samples with higher plugging–inhibitor dosages display a more pronounced increase in viscosity, indicating enhanced structural stability at elevated temperatures. In terms of filtration performance, the experimental results demonstrate that when the concentration of the plugging–inhibitor reaches 1.5 wt%, the API filtration loss (FL) decreases to a minimum value of 6 mL. This observation suggests that the plugging–inhibitor possesses excellent hydration and fluid loss control capabilities. Further analysis reveals that the molecular structure of the plugging–inhibitor contains both adsorption groups and hydration groups. These functional groups can interact with clay particle surfaces through multipoint adsorption, forming a crosslinked network structure. Such a network effectively reduces filtrate invasion and enhances sealing efficiency, thereby contributing to improved overall plugging performance.

#### 3.2.2. Microstructural Morphology of Filter Cake (With Mechanism)

**Figure 6:**
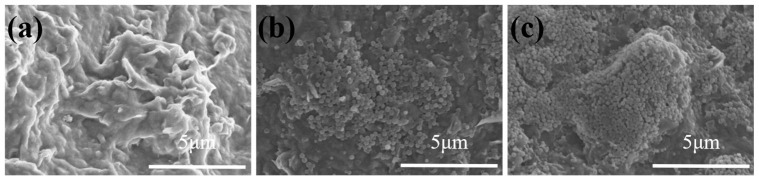
(**a**) Pre-aging slurry, (**b**) pre-aging 1 wt% occlusion-inhibitor, (**c**) pre-aging 2 wt% occlusion-inhibitor.

Figure 6 presents the scanning electron microscopy (SEM) images of the filter cake surface containing DS before aging. As observed, the addition of DS results in significant microstructural changes in the filter cake. A large number of plugging–inhibitor particles are densely packed within the micron-scale pores of the filter cake, effectively occupying the pore spaces. In the regions of shale microfractures, the nanoparticles exhibit pronounced surface adsorption and crack-bridging behavior.

Proposed Mechanism of Interaction:

The DS plugging–inhibitor exhibits amphoteric behavior, containing both NVF-derived groups and anionic AMPS and APS groups. This amphoteric structure enables multisite electrostatic adsorption onto clay surfaces: the NVF-derived groups interact strongly with negatively charged clay particles, while the anionic groups maintain polymer solubility and facilitate the formation of a crosslinked network. As a result, the polymer chains form a three-dimensional adsorption network, which fills nanopores and microfractures, bridges cracks, and enhances the structural integrity of the filter cake. This network not only reduces filtrate invasion but also improves shale inhibition by suppressing clay hydration and swelling. The amphoteric nature of DS is therefore key to its superior plugging and inhibition performance under high-temperature conditions.

**Figure 7:**
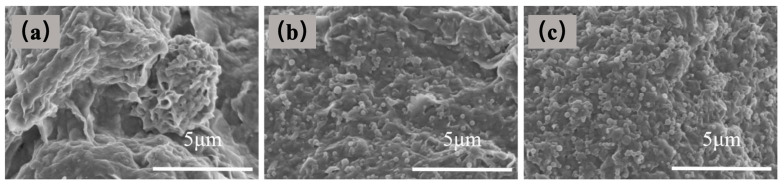
(**a**) Soil slurry after aging, (**b**) 1 wt% occlusion-inhibitor after aging, (**c**) 2 wt% occlusion-inhibitor after aging.

Furthermore, the formation of a three-dimensional network-like sealing layer contributes to improved compactness of the filter cake. Consequently, DS demonstrates an effective nanopore and microfracture plugging capability, consistent with the observed reduction in filtration loss and enhanced structural integrity shown in Figure 6 and Figure 7.

Figure 7 shows the scanning electron microscopy (SEM) images of the filter cake surface containing DS after aging. Compared with the morphology observed before aging, a noticeable reduction in the number of spherical particles is observed after thermal treatment. This change suggests that part of the DS structure undergoes transformation under high-temperature conditions. Meanwhile, the remaining copolymer components that are resistant to thermal degradation effectively fill the pores and fractures within the filter cake. As a result, a more continuous and compact sealing film is formed on the filter cake surface. This film enhances the integrity and density of the filter cake, thereby improving its ability to prevent filtrate invasion. These observations indicate that, even after high-temperature aging, the DS plugging–inhibitor retains its sealing functionality through structural reorganization and pore-filling effects, contributing to sustained plugging performance under harsh conditions.

**Figure 8:**
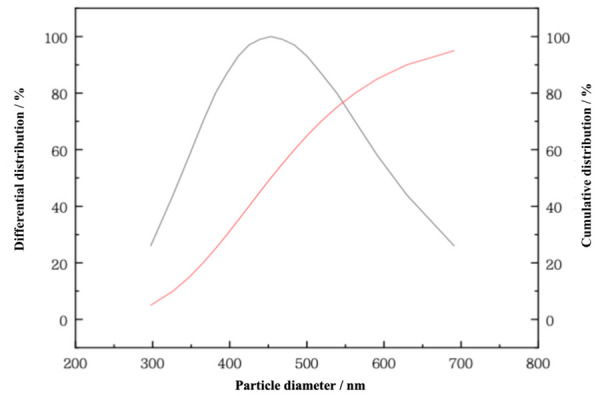
Particle size distribution of the plugging–inhibitor in aqueous suspension measured by DLS. The single-peak distribution with D50 = 453 nm and D90 = 630 nm confirms the micro–nano size of the particles, consistent with SEM results.

In addition, dynamic light scattering (DLS) measurements were performed on the aqueous suspension of the plugging–inhibitor to assess particle dispersion. As shown in Figure 8, the particle size distribution exhibits a single peak with D50 = 453 nm and D90 = 630 nm, confirming that the particle size is in the micro–nano range and consistent with SEM observations. This indicates that the particles are well-dispersed and do not form significant coalesced aggregates.

#### 3.2.3. High-Temperature and High-Pressure Sand Bed Plugging Test

The plugging performance of the plugging–inhibitor was evaluated using a permeability plugging apparatus (PPA) with a sand bed, and compared with that of the base fluid. The sand bed had a pore size of 3 μm, simulating porous media conditions. The experiments were conducted under high-temperature and high-pressure conditions at 180 °C and 3.5 MPa. The filtration volume over 30 min was measured to assess the plugging efficiency of the plugging–inhibitor. The corresponding experimental results are summarized in Table 3. These results provide insight into the effectiveness of the plugging–inhibitor in reducing fluid invasion and enhancing sealing performance under simulated downhole conditions.

The formulation of Base Fluid 2 is 3.0% bentonite + 1.2% fluid loss additive + 0.1% viscosifier + 4% high-temperature dispersant + 4% high-temperature fluid loss reducer + 3% temperature-sensitive asphalt plugging agent + 3% high-temperature anti-sloughing agent + 2% flexible plugging agent (SMFP-2) + 3% micro/nano plugging agent (SMNP-2) + 4% micro/nano plugging agent + 3% extreme-pressure lubricant (SMJH-1) + 3% environmentally friendly lubricant (SMLUB-E) + 7% inorganic inhibitor (ρ = 1.45 g/cm^3^).

The experimental results indicate that the plugging performance of the drilling fluid system is significantly enhanced compared to that of the simple base slurry, with a reduction rate exceeding 80%. A comparison between Base Fluid 1 and Base Fluid 1 with 1% DS plugging–inhibitor, as well as between Base Fluid 2 and Base Fluid 2 with 1% DS, demonstrates that the addition of the DS plugging–inhibitor further reduces the PPA filtration loss for the 3 μm sand bed.

Specifically, in the simple base slurry system, the incorporation of 1 wt% plugging–inhibitor decreases the PPA filtration loss from 24.8 mL to 14.4 mL, corresponding to a reduction rate of 41.94%. In the more complex drilling fluid system, the addition of 1% plugging–inhibitor reduces the PPA filtration loss from 8.8 mL to 6.4 mL, achieving a reduction rate of 28.57%.

These results demonstrate that the DS plugging–inhibitor exhibits an effective plugging capability for porous media with a pore size of 3 μm. Moreover, its performance in both simple and complex drilling fluid systems highlights its adaptability and potential for practical field applications.

### 3.3. Evaluation of High-Temperature Resistance of DS Plugging–Inhibitor

#### 3.3.1. Microstructural Changes in the Plugging–Inhibitor Before and After High-Temperature Aging

**Figure 9:**
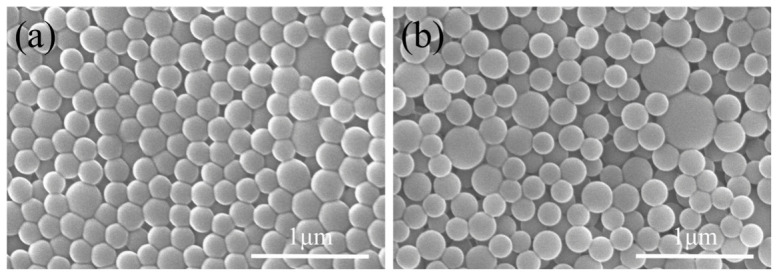
(**a**) Aging at 150 °C plugging and inhibition agent; (**b**) 180 °C plugging and inhibition agent.

A 1 wt% DS plugging–inhibitor emulsion was aged at 150 °C and 180 °C for 16 h. After aging, the samples were deposited onto conductive adhesive, dried, and subsequently characterized using a Regulus-8200 scanning electron microscope to observe the microstructural morphology before and after thermal treatment. The corresponding SEM images are presented in Figure 9.

As shown in Figure 9, the DS plugging–inhibitor maintains excellent dispersion stability under different high-temperature conditions, remaining uniformly distributed in the aqueous system. The particles exhibit regular morphology, predominantly consisting of spherical and flexible structures. Moreover, the particle size distribution remains relatively narrow and uniform.

Notably, even after high-temperature aging, the DS plugging–inhibitor retains a consistent particle size distribution without significant aggregation or structural collapse. These observations indicate that the DS plugging–inhibitor possesses outstanding thermal stability and structural integrity, confirming its suitability for high-temperature applications.

#### 3.3.2. Changes in Plugging Performance Before and After High-Temperature Aging

**Figure 10:**
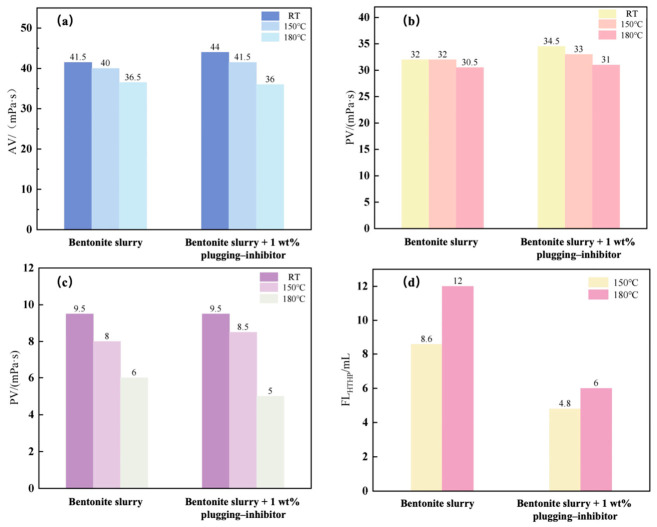
(**a**) Apparent viscosity of drilling fluid before and after different aging temperatures; (**b**) plastic viscosity of drilling fluids before and after different aging temperatures; (**c**) hydraulic shear force of drilling before and after different aging temperatures; (**d**) HTHP filtration of drilling fluids before and after different aging temperatures.

As shown in Figure 10a–c, compared with room temperature, the apparent viscosity and yield point of the drilling fluid containing 1 wt% DS plugging–inhibitor slightly decrease after aging at elevated temperatures (150 °C and 180 °C). However, the magnitude of this reduction is minimal, indicating that the addition of the DS plugging–inhibitor has no significant adverse effect on the rheological properties of the drilling fluid under high-temperature conditions.

The filtration performance of the drilling fluids before and after aging at different temperatures is presented in Figure 10d. It can be observed that the filtration loss increases gradually with increasing temperature. Nevertheless, compared with the base slurry under the same high-temperature conditions, the drilling fluid containing 1 wt% high-temperature-resistant plugging–inhibitor exhibits a significantly reduced filtration loss.

Notably, after aging at 180 °C, the addition of the DS plugging–inhibitor reduces the HTHP filtration loss of the base drilling fluid from 12 mL to 6 mL. This result demonstrates that the DS plugging–inhibitor remains highly effective in controlling filtration loss and forming an efficient sealing barrier even under extreme high-temperature conditions.

The excellent high-temperature plugging performance of the DS plugging–inhibitor can be attributed to its unique molecular structure. The presence of benzene ring structures and sulfonic acid groups contributes to the formation of stable cyclic structures, which enhance the rigidity of the polymer backbone and improve its thermal stability, preventing decomposition at elevated temperatures. In addition, the polymer chains contain highly hydrophilic sulfonic acid groups and functional groups introduced by monomers, which endow the material with strong adsorption capability. The synergistic effect of these structural features enables the DS plugging–inhibitor to maintain effective plugging performance even under harsh high-temperature conditions (Table 4).

### 3.4. Evaluation of Inhibition Performance of DS Plugging–Inhibitor

#### 3.4.1. Linear Swelling Test

**Figure 11:**
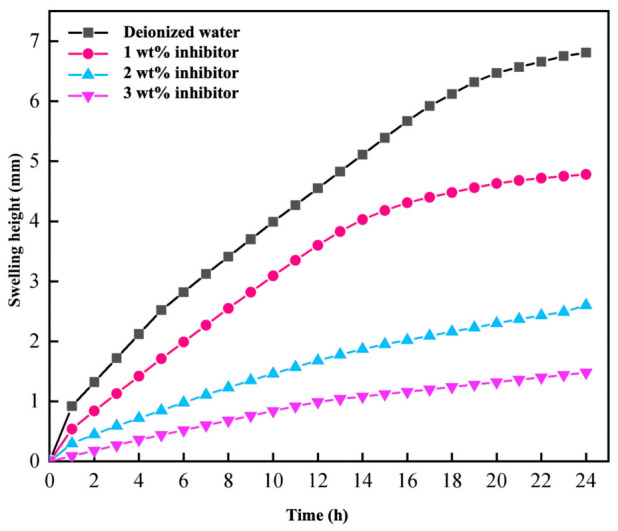
Linear expansion curves of bentonite tablets in different dosage inhibitor solutions.

As shown in Figure 11, the linear swelling height of shale after immersion in deionized water for 24 h reaches 6.81 mm. This swelling behavior of the clay can be mitigated by the DS plugging–inhibitor, which reduces water penetration into interlayer spaces and onto clay surfaces. Hydrogen-bonding interactions between NVF-derived amide groups and hydroxyl sites on clay, combined with hydrophobic segments from styrene and butyl acrylate, and nanoscale polymer particle plugging, collectively suppress hydration-induced swelling of clay particles.

In contrast, when shale samples are immersed in DS plugging–inhibitor solutions with concentrations of 1 wt%, 2 wt%, and 3 wt% for 24 h, the corresponding linear swelling heights decrease to 4.78 mm, 2.6 mm, and 1.48 mm, respectively. These results clearly demonstrate that the plugging–inhibitor exhibits enhanced inhibition performance at higher concentrations.

As the concentration of the plugging–inhibitor increases from 1 wt% to 3 wt%, the swelling height decreases significantly. Notably, the minimum swelling height is observed at a concentration of 3 wt%, indicating that this dosage provides the most effective suppression of shale hydration and swelling.

Furthermore, compared with the deionized water control group, the DS plugging–inhibitor significantly reduces both the swelling rate and the final swelling extent of the shale samples. Specifically, the total swelling is reduced by 77.2% when treated with the DS plugging–inhibitor. These findings provide strong evidence that the DS plugging–inhibitor possesses an excellent hydration inhibition capability.

Although the swelling test was conducted in deionized water, these results, together with mud ball immersion and shale rolling recovery experiments conducted in actual drilling fluids, confirm the inhibitory effect of the DS plugging–inhibitor and its potential practical application.

#### 3.4.2. Mud Ball Immersion Test at Room Temperature

**Figure 12:**
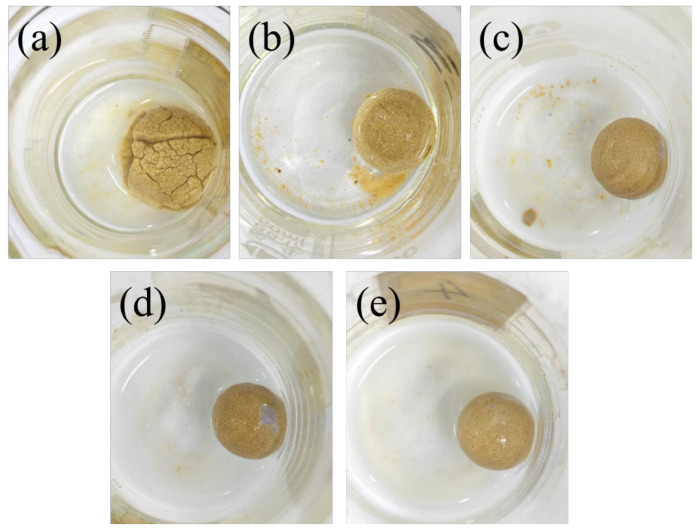
(**a**) The mud bulb was soaked in deionized water, (**b**) 1 wt% NaCl solution, (**c**) 1 wt% inhibitor solution, (**d**) 2 wt% inhibitor solution, (**e**) 3 wt% inhibitor solutionat room temperature.

The morphological changes in mud balls after immersion in deionized water and DS plugging–inhibitor solutions with different concentrations at room temperature for 24 h are presented in Figure 12. The mud ball immersed in deionized water (Figure 12a) exhibits complete disintegration, with evident surface cracking, indicating that the clay particles have undergone extensive hydration and dispersion.

In contrast, when immersed in a 1 wt% NaCl solution (Figure 12b), the mud ball does not show significant swelling; however, surface erosion and particle disintegration are still observed, suggesting that salt alone cannot effectively prevent clay dispersion.

For the samples treated with DS plugging–inhibitor solutions at different concentrations (Figure 12c–e), the mud balls largely retain their original structural integrity. As the concentration of the plugging–inhibitor increases, the surface of the mud balls becomes progressively smoother, and their overall morphology remains more intact. This indicates a stronger inhibition effect against clay hydration and dispersion.

These results demonstrate that the DS plugging–inhibitor can effectively adsorb onto the surface of the mud balls, forming a lower hydrophilic polymer film. This film acts as a barrier to water penetration, thereby preventing water molecules from entering the interior and inhibiting the hydration and dispersion of clay particles. Consequently, the DS plugging–inhibitor exhibits excellent shale inhibition performance.

#### 3.4.3. Mud Ball Immersion Test Under High-Temperature Conditions

**Figure 13:**
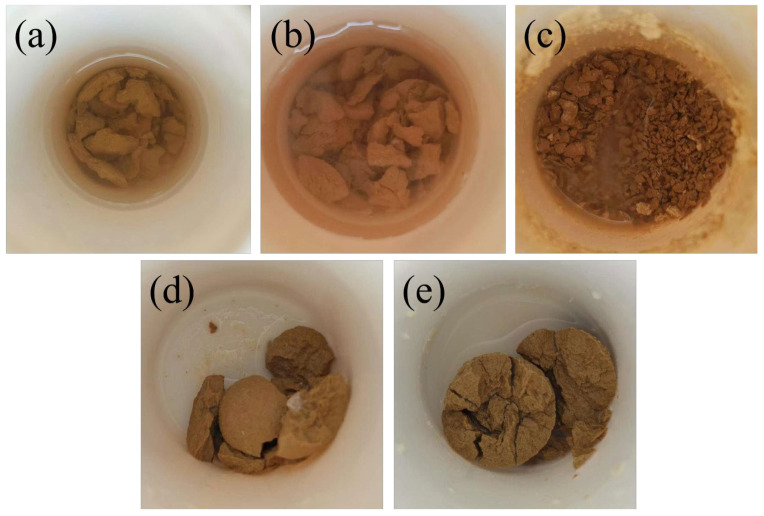
(**a**) Mud balls were soaked at 180 °C for 24 h in deionized water, (**b**) 1 wt% NaCl solution, (**c**) 1 wt% inhibitor solution, (**d**) 2 wt% inhibitor solution, (**e**) 3 wt% inhibitor solution.

The inhibition performance of the DS plugging–inhibitor under high-temperature conditions was systematically evaluated through mud ball immersion tests at 180 °C, with the resulting morphologies shown in Figure 13. After aging at 180 °C for 24 h, the mud balls immersed in deionized water, NaCl solution, and 1 wt% plugging–inhibitor solution were almost completely disintegrated, indicating that their inhibition effects were either negligible or significantly diminished under high-temperature conditions.

In contrast, the mud balls treated with 2 wt% and 3 wt% DS plugging–inhibitor solutions maintained relatively intact structures, clearly demonstrating the enhanced inhibition performance at higher concentrations. These observations suggest that the DS plugging–inhibitor retains an effective inhibition capability under high-temperature conditions when used at a sufficient dosage, highlighting its potential applicability in high-temperature drilling environments.

As shown in Figure 13, the plugging–inhibitor exhibits shale stabilization through the synergistic effects of nanoscale plugging, molecular adsorption, and hydrophobic association. The polymer nanoparticles can penetrate into shale nanopores and microfractures, thereby reducing fluid invasion pathways. Meanwhile, the amide groups originating from NVF are capable of forming hydrogen bonds with hydroxyl groups located on clay mineral surfaces, enhancing polymer adsorption. In addition, the styrene and butyl acrylate segments provide hydrophobic characteristics that reduce water uptake by the shale matrix. These combined effects suppress clay hydration and improve wellbore stability.

#### 3.4.4. Shale Rolling Recovery Test

**Figure 14:**
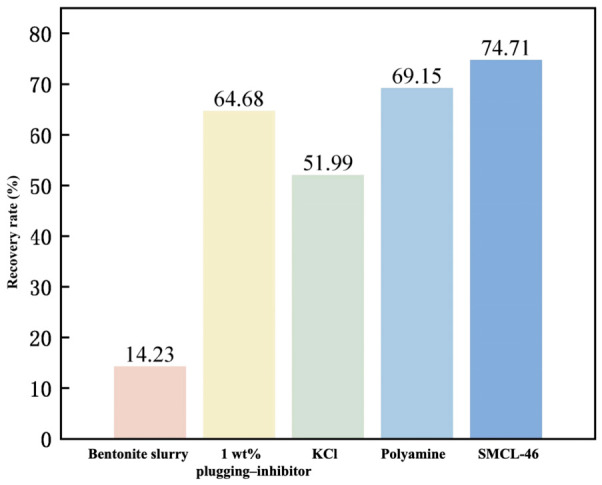
Shale recovery with different inhibitors.

The shale rolling recovery test was conducted to evaluate the hydration dispersion behavior of shale samples. As shown in Figure 14, shale samples were hot-rolled at 180 °C for 16 h in base slurry, 1 wt% DS plugging–inhibitor solution, KCl solution, polyamine solution, and SMCL-46 solution.

The results indicate that the rolling recovery rate of the shale sample in the simple base slurry is only 14.23%, demonstrating severe hydration dispersion and strong water sensitivity. In contrast, the addition of various inhibition agents significantly improves the rolling recovery rate of the shale.

Notably, the drilling fluid containing the DS plugging–inhibitor exhibits a rolling recovery rate of 64.68%, representing a substantial improvement compared to the base slurry. This result confirms that the DS plugging–inhibitor provides strong inhibition against shale hydration and dispersion, highlighting its effectiveness as a shale stabilizing agent under high-temperature conditions.

The improved shale recovery performance is mainly attributed to the adsorption and sealing effects of the polymer nanoparticles. Hydrogen-bonding interactions between the polymer amide groups and clay mineral surfaces promote the formation of a protective layer, while the hydrophobic polymer segments reduce water penetration into the shale. Simultaneously, the nanoscale particles effectively seal nanopores and microfractures, thereby minimizing shale hydration and dispersion.

## 4. Conclusions

A high-temperature-resistant DS plugging–inhibitor was successfully synthesized via emulsion polymerization using BA, St, AMPS, and NVF as monomers, OP-10 as the emulsifier, DVB as the crosslinker, deionized water as the dispersion medium, and APS as the initiator. The structure, thermal stability, morphology, and wettability of the synthesized material were systematically characterized by FT-IR, TG, SEM, and contact angle analysis.

The results show that the DS plugging–inhibitor exhibits a regular spherical morphology with a particle size distribution of 50–100 nm and lower hydrophilicity. Thermogravimetric analysis indicates that the main decomposition temperature is approximately 297.07 °C, demonstrating excellent thermal stability.

Filtration and plugging performance evaluations, including API filtration and high-temperature high-pressure (HTHP) sand bed plugging tests, confirm that the DS plugging–inhibitor effectively reduces filtration loss and exhibits strong plugging capability. SEM analysis of filter cakes reveals that the plugging–inhibitor can efficiently fill pores and form a dense sealing structure, thereby reducing fluid invasion into the formation.

The inhibition performance was further evaluated through mud ball immersion tests, linear swelling tests, and shale rolling recovery tests. The results demonstrate that the DS plugging–inhibitor exhibits excellent inhibition performance. At a dosage of 3 wt%, the linear swelling height is reduced to 1.48 mm, and the mud ball maintains good structural integrity. Additionally, the rolling recovery rate reaches 64.68% at 180 °C, indicating strong inhibition and high-temperature resistance.

The excellent plugging and inhibition performance of the synthesized polymer is attributed to the combined effects of nanoscale pore sealing, hydrogen-bonding adsorption, hydrophobic association, and the formation of a dense protective film on shale surfaces. These mechanisms effectively reduce fluid invasion and suppress shale hydration, thereby enhancing wellbore stability under high-temperature conditions.

In summary, the DS plugging–inhibitor exhibits excellent thermal stability, plugging performance, and shale inhibition capability, making it a promising candidate for application in high-temperature water-based drilling fluids.

## Figures and Tables

**Table 1:** Thermogravimetric data for occlusion-inhibitor DS.

Sample	T_10_% (°C)	T_30_% (°C)	T_50_% (°C)	Residual Carbon (%)
Plugging–inhibition agent	307.1	346.1	362.1	1.776

**Table 2:** Rheological properties before and after aging at 150 °C for 16 h.

No.	Formulation	Test Condition	AV (mPa·s)	PV (mPa·s)	YP (Pa)	FL_API_ (mL)
1	4% Bentonite Slurry	Before rolling	14.5	7.5	14.3	12
		After rolling	4.5	5	13.3	30
2	4% Bentonite Slurry + 0.5% Plugging Agent	Before rolling	15	7	15.8	8
		After rolling	4	5.5	12.8	26
3	4% Bentonite Slurry + 1% Plugging Agent	Before rolling	15.5	7.5	16.9	7
		After rolling	5	6	15.8	25
4	4% Bentonite Slurry + 1.5% Plugging Agent	Before rolling	16.5	8	17.4	6
		After rolling	5.5	6	14.8	23
5	Base Fluid 2 (freshwater-based bentonite slurry)	Before rolling	7.5	7.5	14.3	–
		After rolling	7.3	7.2	13.9	–

**Table 3:** Occlusion-inhibitor occlusion evaluation results.

No.	Formulation	PPA Filtration Loss (mL)	Reduction Rate (%)	HTHP (mL)
1	Base Fluid 1	24.8	—	Total loss
2	Base Fluid 1 + 1% Plugging Agent	14.4	41.94	10.4
3	Base Fluid 2	8.8	38.89	8.8
4	Base Fluid 2 + 1% Plugging Agent	6.4	28.57	8.6

Note: The formulation of Base Fluid 1 is 4.0% bentonite slurry + 0.3% PAC-LV.

**Table 4:** Evaluation of rheological properties and filtration performance of drilling fluids before and after aging at different temperatures.

Formulation	Test Condition	AV (mPa·s)	PV (mPa·s)	YP(Pa)	FLHTHP (mL)
Base Fluid	Room temperature	41.5	32	9.5	–
	150 °C	40	32	8	8.6
	180 °C	36.5	30.5	6	12
Base Fluid + 1% Plugging–Inhibitor	Room temperature	44	34.5	9.5	–
	150 °C	41.5	33	8.5	4.8
	180 °C	36	31	5	6

## Data Availability

The data that support the findings of this study are available from the corresponding author due to privacy.
